# How Many Chemicals in Commerce Have Been Analyzed in Environmental Media? A 50 Year Bibliometric Analysis

**DOI:** 10.1021/acs.est.2c09353

**Published:** 2023-06-15

**Authors:** Derek C. G. Muir, Gordon J. Getzinger, Matt McBride, P. Lee Ferguson

**Affiliations:** † Environment & Climate Change Canada, Burlington, Ontario L7S1A1, Canada; ‡ School of Environmental Sciences, University of Guelph, Guelph, Ontario N1G2W1, Canada; § School of Environmental Sustainability, Loyola University Chicago, Chicago, Illinois 60660, United States; ∥ Department of Civil and Environmental Engineering, Duke University, Durham, North Carolina 27708, United States; ⊥ CAS IP Services, CAS, Columbus, Ohio 43202, United States

**Keywords:** bibliometric survey, chemical inventories, analytical study, pollutant, environmental measurement, citation counts

## Abstract

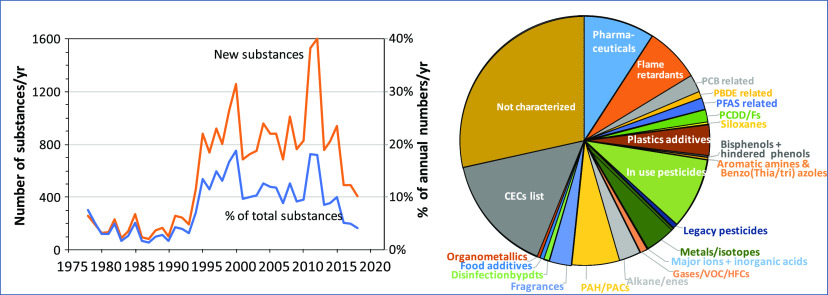

Over the past 50 years, there has been a tremendous expansion in the measurement of chemical contaminants in environmental media. But how many chemicals have actually been determined, and do they represent a significant fraction of substances in commerce or of chemicals of concern? To address these questions, we conducted a bibliometric survey to identify what individual chemicals have been determined in environmental media and their trends over the past 50 years. The CAplus database of CAS, a Division of the American Chemical Society, was searched for indexing roles “analytical study” and “pollutant” yielding a final list of 19,776 CAS Registry Numbers (CASRNs). That list was then used to link the CASRNs to biological studies, yielding a data set of 9.251 × 10^6^ total counts of the CASRNs over a 55 year period. About 14,150 CASRNs were substances on various priority lists or their close analogs and transformation products. The top 100 most reported CASRNs accounted for 34% of the data set, confirming previous studies showing a significant bias toward repeated measurements of the same substances due to regulatory needs and the challenges of determining new, previously unmeasured, compounds. Substances listed in the industrial chemical inventories of Europe, China, and the United States accounted for only about 5% of measured substances. However, pharmaceuticals and current use pesticides were widely measured accounting for 50–60% of total CASRN counts for the period 2000–2015.

## Introduction

The increased emphasis on “chemicals of emerging concern” since the early 2000s,^1^ and the more recent growth of nontarget screening,^2,3^ is broadening the list of chemicals identified and measured in environmental media. But how many substances have actually been determined in environmental media, and are they representative of the growing numbers of chemicals in commerce? A survey of the chemical inventories of 19 countries and regions revealed that about 350,000 chemical substances have been registered for production and large scale use over the past 30 to 40 years.^4^ About 700 new chemicals per year are officially added to the US Toxic Substances Control Act (TSCA) inventory each year,^5^ while the European Chemicals Agency has registered about 1700 (new and existing substances) per year under Registration, Evaluation, Authorization and Restriction of Chemicals (REACH) since 2009.^6^ The threshold for registration under REACH and for the Inventory of Existing Chemical Substances of China (IECSC) is 1 t/yr, while it is 11 t/yr under TSCA. About 4600 chemicals are considered to be high production volume (>1000 t/yr).^7^ Globally approximately 4000 human and veterinary pharmaceuticals,^8^ 1800 pesticides,^9^ and 4000 fragrance ingredients^10^ have been registered under national or regional Food and Drug and pesticide use legislation. Altogether the emissions from production, use, and recycling of commercial chemicals, combined with their byproducts and transformation products, represent a very large set of possible compound leads for “suspect screening” or targeted analysis of environmental samples.^3^

However, bibliometric surveys suggest that only a limited number of chemicals have been determined in environmental media. For example, in an analysis of nearly 120,000 articles in environmental science and toxicology published over the period 2000 to 2009, Grandjean et al.^11^ found that just 100 substances (metals, polycyclic aromatic hydrocarbons (PAHs), polychlorinated biphenyls (PCBs), pesticides) represented 24% of all compounds reported. Similarly, Daughton^12^ found that the majority of environmental measurements of pharmaceuticals focused on <100 active ingredients. Sobek et al.^13^ showed that of the 105 different substances analyzed in Baltic Sea fish, the majority of analyses (87%) were for 20 substances. In a survey of exposure data on the USEPA ACTOR database (now part of Chemicals Dashboard; https://comptox.epa.gov/dashboard), Egeghy et al.^14^ found that, out of 547,000 substances, available environmental measurement data were very limited with water having the largest number of unique compounds (1150 chemicals). Halden^15^ examined progress in the analysis of chemicals of emerging concern (CEC) based on 12 substances using a comprehensive literature search with SciFinder software and noted a common pattern of emergence of publications, which was related to new analytical methods, regulatory actions, and mass consumption of novel products.

The studies by Grandjean et al.,^11^ Daughton,^12^ and Sobek et al.^13^ all point to a bias toward repeated measurements of the same compounds and a lack of studies on a broader suite of substances. Grandjean et al.^11^ termed this bias the “Matthew Effect”, using a term originally used by Merton^16^ to describe the heightened “visibility of contributions to science by scientists of acknowledged standing”. In the context of environmental contaminants, this bias may reflect a tendency to avoid the risks associated with unstudied chemicals such as the lack of analytical methods and standards as well as the desire for more knowledge of toxicity and/or physical chemical properties before committing laboratory resources or changing monitoring targets. In a bibliographic assessment of the citations of his own publications over a 50 year career, Professor Ron Hites^17^ has pointed out that compounds that are relatively easy to measure, e.g. brominated flame retardants, have received more attention than those that require new methods or instrumentation. Hites^17^ also noted that toxicity and environmental persistence of a compound or class of substances are generally major drivers of interest.

The bibliometric surveys related to the question of what chemicals have been measured to date in environmental media have been limited in terms of time window or chemicals of interest. Also, previous surveys may not have captured the growing interest in CECs and in nontargeted analysis. We believe that a more comprehensive assessment is needed to further illuminate the topic and act as a guide for future chemical screening. Our objectives were to (1) Identify what individual chemicals have been determined in environmental media using appropriate search terms, (2) Assess the “Matthew effect” by comparing total substance “hits” each year with numbers of substances reported for the first time, and (3) Compare substances with lists of inventoried or priority chemicals to assess progress in determining environmental occurrence of chemicals in commerce. To accomplish this, we utilized a bibliometric search approach similar to Halden^15^ for papers compiled in the CAS (CAplus) database.

## Methods

The CAplus database (http://support.cas.org/content/references) was searched using the CAS indexing term “Role” that describes the new or novel information reported about a substance or a class of compounds. We selected the Roles “analytical study (ANST)”, “pollutant (POL)”, and “biological study (BIOL)” to identify the individual chemicals and to determine the number of citations for each one over time. These three “Roles” are defined in more detail in the Supporting Information. We used two complementary approaches:

1. The CA indexing roles ANST and POL were used to search the CAplus database for CAS registry numbers (CASRNs) indexed with these roles in papers published over the period 1978–2018. These “Roles” were judged to be sufficiently comprehensive to capture substances detected or identified in environmental media while omitting toxicology studies, chemical synthesis, or use as chemical reagents, etc. Patents were excluded. CASRNs associated with “analytical study” and “pollutant” were generated for each year and thus contained many duplicates as many of the substances were reported each year. A final list of CASRNs was created by removing duplicates sequentially starting with the earliest year using a duplicate finder (AbleBits.com). This is referred to hereafter as the ANST/POL list or search. It was completed in April 2018. After reviewing the substances in the ANST/POL list it was further reduced as discussed below.
2. Using the indexing Role “BIOL” (biological study) for citations of the CASRNs identified by the ANST/POL search, for the period 1966 to 2021: This was judged to be a search strategy that would yield a more representative total number of citations for each substance identified in #1 as well as confirm actual environmental measurements. The BIOL role was assigned to a substance in studies of the role of the substance in or its effect on biological molecules include metabolism, toxicity, occurrence, biological applications, and composition.^18^ This is referred to hereafter as the BIOL/Occur list.

InCHI and SMILES codes, along with chemical names, were generated for the full list of CASRNs in the ANST/POL list. This was done with CAplus and also using various online databases including the EPA Chemical Dashboard (https://comptox.epa.gov/dashboard), Chemical Translation Service (http://cts.fiehnlab.ucdavis.edu), OPSIN (https://opsin.ch.cam.ac.uk/), and NCI/CADD Chemical Identifier Resolver (https://cactus.nci.nih.gov/chemical/structure). Structural characteristics of about 900 CASRNs could not be assigned from database searches and were reviewed manually using PubChem and SciFinder searches as they were mixtures, trade names, polymers, proteins, or otherwise undefined. About 300 partially defined substances were assigned representative SMILES and InChI by drawing most likely structures (e.g., bromo-, methoxy diphenyl ethers, bromo-chloro carbazoles, chloro alkanes, alkylated PAHs, PCBs (as Aroclors, Phenoclor, Kanechlors) or polybrominated diphenyl ethers (Bromkal). The final list is provided in the Supporting Information as an Excel Spreadsheet.

CASRNs in the BIOL/occur list were categorized by searching lists of known substances including perfluorinated compounds, pharmaceuticals, pesticides, flame retardants, fragrances, bisphenols and other plastics additives, trace gases, metals, and major inorganic ions available on the US EPA CompTox Chemicals Dashboard and other Web sites (Table S1). By “class” we mean substances with similar chemical structures (e.g., bisphenols) and/or function or purpose in society (e.g., pharmaceuticals, pesticides). The common names were also screened manually for related substances such as transformation products of pesticides, PCBs, PBDEs, pharmaceuticals, and polycyclic aromatic compounds. The citations per year of the CASRNs within each class in the BIOL/occur list were determined. The individual chemicals on the ANST/POL list, and their annual citation counts per year, were also compared with publicly available lists of chemicals on the chemical inventories of the US (TSCA), EU (REACH), and China (IECSC).

## Results and Discussion

### 1 Overview of Occurrence

A total of 105,410 citations were found using the roles “analytical study” and “pollutant” for the period January 1978–April 2018 based on 23,458 unique CASRNs (Table 1; See also further discussion of citation counts in the SI). The citation reports represented 468,383 citation counts of the CASRNs due to multiple CASRNs reported in many published articles. Review of the total counts for the most reported CASRNs showed that they included essential elements (e.g., calcium), inorganic salts, minerals, many natural products, and substances with complex or no structures (Table S2A, B). Also present were natural food related constituents e.g. glucose, lactose, sucrose. To improve the focus on CASRNs more likely to be labeled contaminants or pollutants, we manually screened the ANST/POL list and removed 3,682 CASRNs. The categories removed are shown in Table S2A. They included acids, alloys, complex structures (e.g. DNA, proteins, enzymes), mass labeled (^13^C, ^2^H, ^15^N) compounds, minerals, most simple inorganic salts except well-known water pollutants (perchlorate, nitrate, phosphate, sulfate, cyanide ion), 9 essential elements, polymers, natural products, mixtures, and CASRNs which CAplus labeled “Index Name Not Yet Assigned” and for which no structural information was available on SciFinder or via a Google search. We also used the FooDB^19^ database of food constituents to select 922 CASRNs for removal as they appeared to be natural constituents. The final ANST/POL list comprised 19,776 CASRNs with 360,247 total counts or citations (Excel file, Table S3).

**Table 1:** Numbers of Substances Found with the “ANST/POL” and “BIOL/Occur” Searches along with Total Reports, Total Counts, and Percentage of the Total Represented by the Top 100 to 500

	ANST/POL search			BIOL/Occur search		
Search/with number of CASRNs used	Total CASRN citations^a^	Total CASRN counts^b^	% of total	Total CASRN citations^a^	Total CASRN counts^b^	% of total
Initial list of CASRNs (23,458)	105,410	468,383		479,636	21,828,774	
Final CASRNs (19,776)	89,959	413,007		359,641	9,251,975	
Top 10		36,128	8.8%		950,454	10%
top 100		140,527	34%		3,303,278	36%
top 200		190,759	47%		4,469,288	48%
top 300		222,788	54%		5,214,655	56%
top 500		262,875	64%		6,166,490	67%
Lead		6,224	1.5%		73,043	0.79%
Cadmium		4,692	1.1%		82,544	0.89%
p,p′-DDT		1,571	0.38%		15,534	0.17%
2,3,7,8-TCDD		743	0.18%		10,066	0.11%

a Total citation counts of CASRNs for all years of the search window (Jan 1978–Apr 2018 for ANST/POL and Jan 1966–Dec 2021 for BIOL/Occur). Note that the BIOL/Occur search used the ANST/POL CASRN list (Table S4).

b Total citation counts for each CASRN over the search window (1978–2018 for ANST/POL and 1966–2021 for BIOL/Occur).

The BIOL/Occur search using the reduced ANST/POL list had 9,251,975 total counts (Table 1) indicating that the search using BIOL (biological study) as a CA Role captured a much wider array of reports due to including the roles such as biological effect and metabolism as well as occurrence. Nevertheless, we chose to use the term BIOL/Occur to distinguish this group.

The most repetitively analyzed CASRNs appearing in the ANST/POL search were heavy metals (lead, cadmium, and copper), gaseous pollutants (sulfur dioxide, nitrogen dioxide, carbon monoxide, and carbon dioxide), and aromatics including PAHs (benzene, toluene, and benzo(a)pyrene) (Table S4). The top 100 substances represented 34% of the total counts (413,007) for the 19,776 CASRNs rising to 64% for the top 500. The BIOL/Occur search with the same CASRNs brought a different suite to the top of the list, with highest counts for copper, magnesium, nitrogen oxide, and carbon dioxide along with nutrients nitrate and phosphate (Table S4). Although the BIOL/Occur search shifted the counts toward a biomedical focus, it nevertheless did capture larger counts for known pollutants. For example, lead increased from 6,224 to 73,043 counts, and 2,3,7,8-TCDD increased from 743 counts to 10,066 (Table 1). Thus, while the ANST/POL search appeared to have captured a very large range of chemicals, the full number of reports and citations for these chemicals was better reported with the BIOL/Occur role.

**Figure 1:**
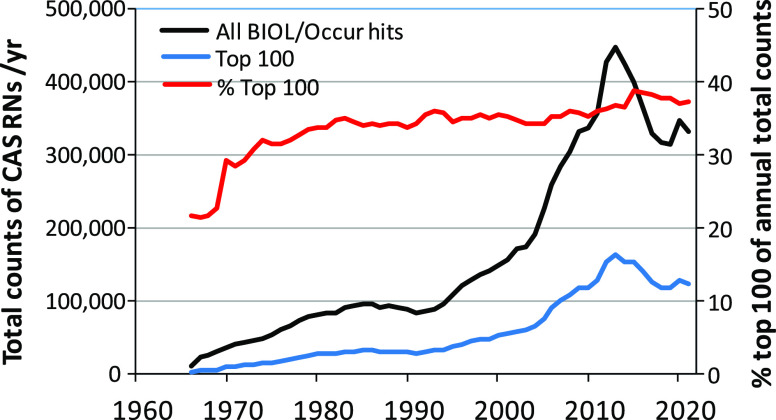
Trends of total counts for 19,776 CASRNs using the BIOL/Occur search in CAplus compared with the counts for the top 100 substances. The % represented by the top 100 is shown as a red line using the right-hand axis.

The total number of published reports for chemicals in the BIOL/Occur search was relatively steady during the 1980s (median 1980–1989 of 89,500) but rose 5-fold from 1993 to 2013 (Figure 1). A relatively small number of substances dominated the total counts (Table 1). This result is similar to what other studies have found. Grandjean et al.^11^ reported 20 substances accounted for 12% of all CAS number links for the period 2000–2009. Here, the top 10 most repetitively analyzed chemicals using the ANST/POL list were lead, cadmium, copper, chromium, arsenic, mercury, and nickel along with benzene and toluene; they accounted for 8.8% of all reports. For the BIOL/Occur list, which was heavily influenced by biomedical literature as discussed below, the top 10 shifted to copper, magnesium, nitrogen oxide, manganese, cadmium, carbon dioxide, lead, hydrogen peroxide, lactic acid, and nitrate, accounting for 10.4% of the 9.251 × 10^6^ reports (Table 1). The top 100 substances accounted for 34% of 19,776 substances using the ANST/POL search and 35% using the BIOL/Occurrence search. The percent of the total counts of CASRNs per year that these 100 substances represented gradually increased over the period 1966 (22%) to 2016 (37%) (Figure 1). This reflects the fact that many of these are well-known pollutants and toxic substances that continue to be measured in biological and environmental media. It should be noted that the search did not consider whether the substances were above detection limits, only that they were in the analytical list of the published article within the CAplus database.

**Figure 2:**
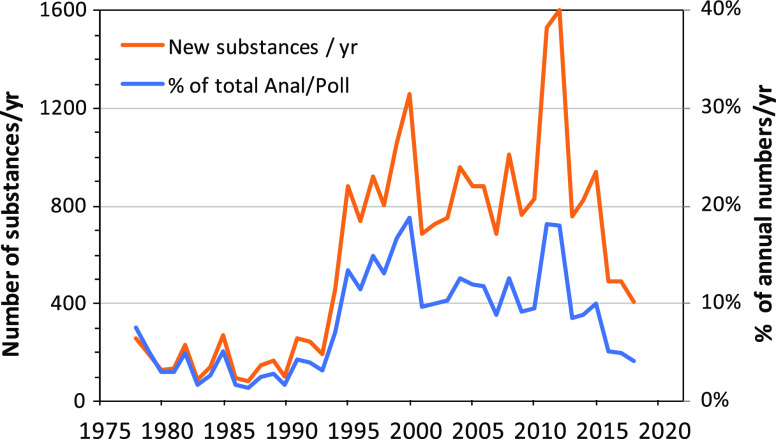
Trends of the number of newly reported substances using CA indexing roles “analytical study” and “pollutant” (ANST/POL) CASRNs for the period 1978–2018 (left vertical axis) and as a percentage of the total number of reports per year for all 19,776 substances in the reduced ANST/POL list based on the BIOL/Occurrence search (right vertical axis).

The number of substances reported for the first time each year based on the ANST/POL search reached a maximum of 1600 in 2012 (Figure 2). It is reported only until 2018 because our original search was conducted in April 2018. A sharp jump occurred in the mid-1990s and reached a maximum of 18% of the total number of CASRNs in the ANST/POL list in 2012.

### 2 Comparison with Chemical Lists

**Figure 3:**
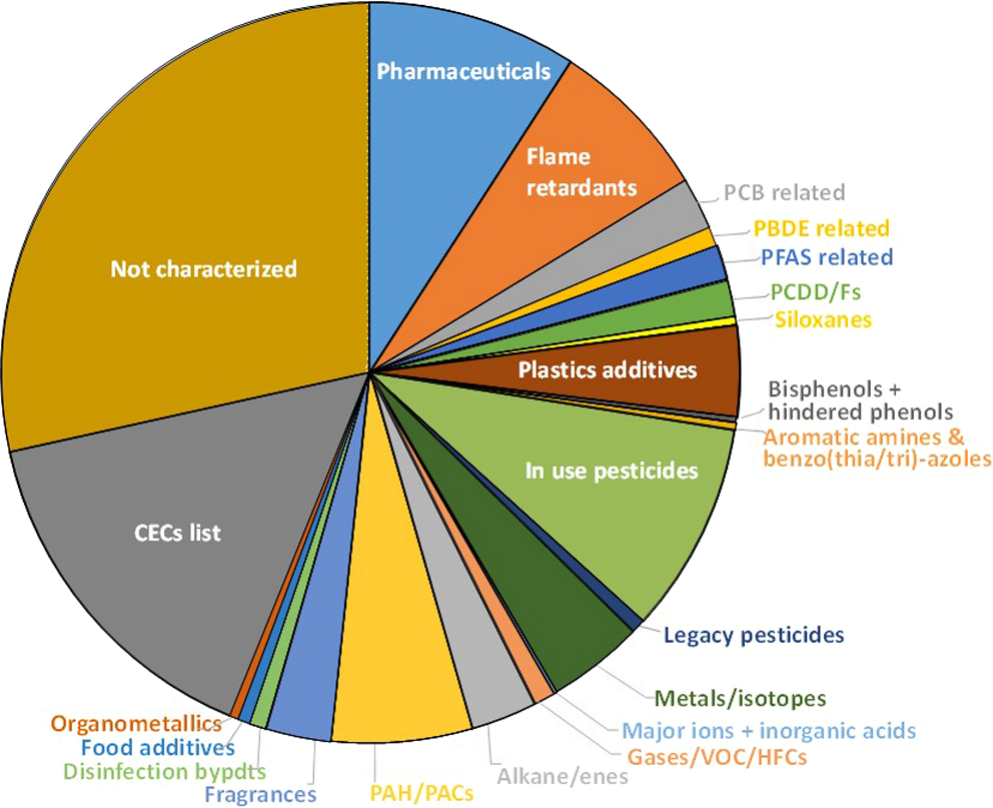
Categorization of the ANST/POL list of 19,776 CASRNs based on screening of lists for various classes of substances (see Table S1). The “Not characterized” class represents a diverse group not found on lists of various monitoring and priority pollutant lists.

The ANST/POL list was screened for known chemicals using lists from the USEPA CompTox Chemicals Dashboard and other publicly available lists (Table S1). In total, 14,152 CASRNs (71% of the ANST/POL list) were found based on the substances in these lists as well as their analogs and transformation products. The latter were generally not included in the lists and had to be identified manually. The distribution among 22 major classes is shown in Figure 3 and presented in greater detail in Table S2B. Most of these classes of substances are well-known environmental contaminants (PCBs, legacy organochlorine (OC) pesticides, PBDEs, PAHs, in use (or current use) pesticides, and pharmaceuticals) although some such as plastics additives, bisphenols and siloxanes, have more limited measurements as discussed below. The list of known chemicals on the ANST/POL list included 3041 substances (17%; Figure 3) that were found only on the large list of CECs published by Meijer et al.^20^ and also available in the NORMAN network SUSDAT list (Table S1). That list (∼62,260 CASRNs as of June 2020) includes a wide range of chemistries plus predicted Phase 1 degradation products. The remaining 29% represents a diverse group not found on lists of various monitoring and priority pollutant lists although all were easily represented structurally with InChI codes.

The 14,152 substances on lists paralleled the total BIOL/Occur counts closely (Figure S1A) averaging 92% (range 91–94%) of all CASRN reports for the ANST/POL list over the period 1966 to 2021 (Figure S1B). Individual classes within the 14,152 varied substantially over the same period, as discussed below, but this diverse group as a whole represents the majority of chemicals monitored in environmental media. The 3041 CECs from the SUSDAT list represented an additional 3.2 to 6.3% of the CASRN counts/year, while the CASRNs not found in lists was relatively constant averaging 7.6% of reports (Figure S1A and S1B).

Pharmaceuticals represented the largest easily categorized class within the ANST/POL list (Figure 3; Table S2B). The 1809 substances (including transformation products) identified from lists and by manual searches was greater than the number anticipated based on recent reviews of environmental measurements. A very comprehensive list of pharmaceuticals measured in environmental media^21^ is available from UBA (https://www.umweltbundesamt.de/en/database-pharmaceuticals-in-the-environment-0). It contains about 849 pharmaceuticals and transformation products of which 615 are present in the ANST/POL list. However, the ANST/POL list contains another 1200 substances that are listed as pharmaceuticals or their transformation products based on a combined list of about 7383 CASRNs (Table S1). The top 5 compounds in the UBA database are ibuprofen, diclofenac, carbamazepine, sulfamethoxazole, and trimethoprim, while the top 5 in the ANST/POL database are cisplatin, dexamethasone, ciprofloxacin, gentamicin, and doxorubicin (Table S5). This striking difference reflects the biomedical science influence of the BIOL/occur list. A search of SciFinder (using the “Occurrence” role and then categorizing for environmental chemistry and pollutants) found published articles for all 10, with better overall agreement with the studies on the UBA list. Based on this subset, it is apparent that the BIOL/occur role appears to significantly overestimate the pharmaceuticals measured in environmental media due to inclusion of the extensive number of biomedical related studies which involve analysis, such as clinical measurements and antibiotic resistance, but not pollution.

### 3 Temporal Trends of Major Individual Classes

**Figure 4:**
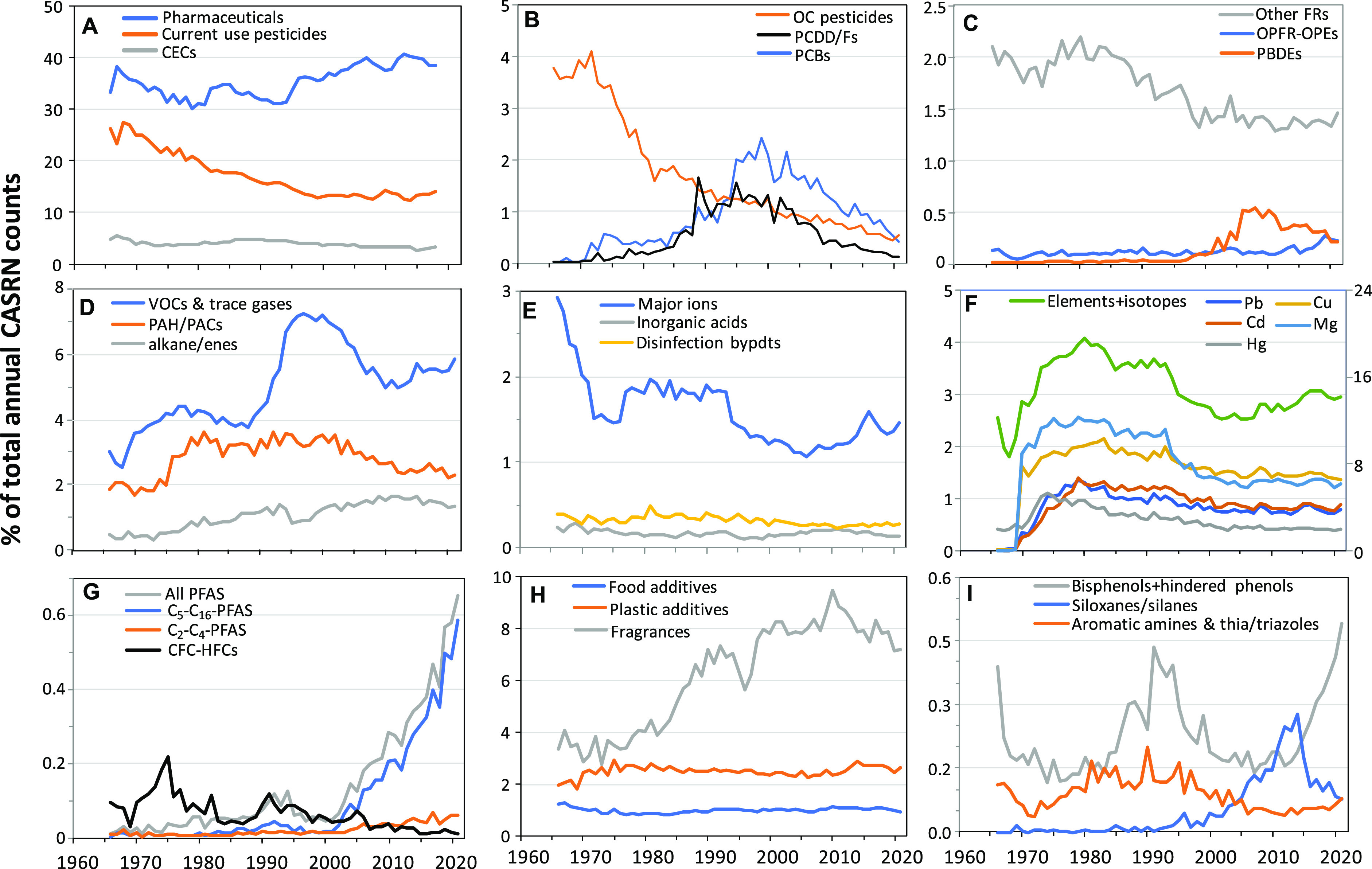
Percent of total annual CASRN reports for (A) pharmaceuticals, current use pesticides, CECs not on other lists, (B) Legacy organochlorine (OC) pesticides, polychlorinated dibenzo-p-dioxins and -dibenzofurans (PCDD/Fs), polychlorinated biphenyls (PCBs), (C) polybrominated diphenyl ethers (PBDEs), organophosphate flame retardants and phosphate esters (OPFR-OPEs), other flame retardants (FRs), (D) Volatile organic compounds and trace gases, polycyclic aromatic hydrocarbons/compounds (PAHs/PACs), alkane/enes, (E) Major water pollutant ions, inorganic acids, disinfection byproducts, (F) Metal isotopes and major metals (scale on the right), copper (Cu), magnesium (Mg), cadmium (Cd), lead (Pb), mercury (Hg), (G) poly/perfluoroalkyl substances (PFASs) including chlorofluorocarbons and hydrofluorocarbons (CFCs-HFCs), (H) Food additives, plastics additives, fragrances, (I) Bisphenols, siloxane/silanes, aromatic amines, benzotriazoles/benzothioazoles. The CASRNs also include transformation products for all classes. See Figure S4 for CASRN counts/year.

Trends of 25 classes of substances over time based on the BIOL/Occur counts of reports of CASRNs are shown in Figure 4 as a percent of total reports and in Figure S2B as total counts of CASRNs reported each year. Pharmaceuticals, current use pesticides, and CECs are combined in Figure 4A because they represent the largest classes within the BIOL/occur search accounting for 50 to 82% of total CASRN counts over the period 1966 to 2018. Pharmaceuticals and pesticides are often viewed as CECs;^22,1^ however, here CECs are more narrowly defined as substances not present on other lists (Table S1) but included in the CEC screening list of Meijer et al.^20^ and in SUSDAT. Reports of pharmaceuticals began a sharp upward increase around 1990 and increased 6-fold to a maximum in 2013 (Figure S2A). From 1980 this group represents the largest percentage of total CASRNs reported per year for any of the classes listed in Table S5, reaching 42.5% of the total counts in the BIOL/Occur search (Figure 4A). The 615 CASRNs on the UBA database increased from 0.1% to 8.3% of total CASRN counts in 1998 and 2008, respectively (Figure S3). This reflected a rapid increase in environmental measurements as discussed by aus der Beek et al.^21^

Current or in-use pesticides (i.e. those not currently banned by various national/regional regulatory actions or under the Stockholm Convention) represented 9.2% of the BIOL/occur list (Figure 3). After being relatively constant from 1975 to 1990, reports of pesticide CASRNs increased 4-fold from 1990 to a maximum in 2012 (Figure S2A). However, as a percent of total annual counts of pesticide CASRNs have declined from 25% in 1970 to 14% in 2021, reflecting the rise in other classes, especially pharmaceuticals (Figure 4A). Lindane and chlorpyrifos represented the most widely measured current use pesticides with 12386 and 11828 reports, respectively, over a 50 year period (Table S4).

The CECs from the SUSDAT list changed very little in terms as a proportion of reported total annual CASRNs ranging from 4.0% in 2001 to 3.6% in 2021 (Figure 4A). The CECs are not new substances as most of the CASRNs have reports over a 50+ year period. This diverse group included 459 halogenated aromatics and 1138 substances that had monocyclic aromatic moieties but were not halogenated. The haloaromatics ranged from 5.2 to 10%, and the nonhalogenated aromatics ranged from 33 to 51% of the 3041 substances from 1966 to 2021 (Figure S4).

PCBs, PCDD/Fs, and OC pesticides represent classes of substances which have been widely measured in environmental media since the late 1980s or earlier in many cases, as part of regional, national, and global research and monitoring programs. Reports for PCBs, including hydroxyl- and methylsulfone degradation products, reached a maximum of 2.4% of BIOL/occur results in 1999 (Figure 4B), and numbers of reports of PCB CASRNs remain relatively high in the 2000s, reaching a maximum of 4933 in 2008 (Figure S3). The continued interest in PCBs reflects their presence on priority chemical lists and also increased interest in byproduct PCBs emitted from paint pigments and other non-Aroclor sources.^23,24^ PCDD/Fs, and their transformation products, had a similar temporal pattern as PCBs, reaching a maximum number of reports in 2007 (1.1% of total CASRN reports) and declining after 2010. Chlorinated pesticides (DDT, chlordane, dieldrin, toxaphene, etc.) and their transformation products declined from a high of 4.3% of BIOL/occur results in 1972 to 0.4% in 2020. However, the period 2000–2012 saw a doubling of the number of reports of legacy OC pesticides (Figure S3) which is part of an overall increase in published reports for all CASRNs on the ANST/POL list (Figure 1).

Flame retardants (FRs) represented 1.5 to 2.3% of BIOL/occur results from 1966 to 2021 (Figure 4C). Our list was based on the USEPA “FLAMERETARD” with additions of polybrominated diphenyl ethers (PBDEs), bromophenols, bromo/chlorobenzenes, polychlorinated diphenyl ethers (PCDEs), chlorinated polycyclic aromatics (e.g. Dechloranes), and chlorinated paraffins. PBDEs represented a major fraction of the FRs from 2000 to 2015, reaching a maximum of 25% of reports for FRs in 2008. OPFR-OPEs increased after 2010 reflecting their increasing use as replacements for PBDEs.^25,26^ The FRs were a diverse class with substances having no chlorine, bromine, or phosphorus (e.g. melamine, borates, etc.) constituting the largest group followed by various chlorinated compounds (Figure S5A). Reports of the nonhalogenated FRs declined from 1980 to 2000 but have increased to 1.2% of CASRN counts in 2021, while non-PBDE brominated FRs remained relatively steady at 0.14 to 0.21% from 2000 to 2021 (Figure S5B).

PAHs including alkylated and polycyclic aromatic compounds (PACs) varied between 1.7 and 3.6% of total CASRN counts over most of the time period (Figure 4D). Major PAH/PACs were unsubstituted priority compounds benzo[a]pyrene, naphthalene, phenanthrene, pyrene, and anthracene (Table S4). Two alkylated PAHs, 3-methylcholanthrene and 7,12-dimethylbenz(a)anthracene, were among the 20 PAHs in the top 500 analytes, possibly because of extensive studies on their carcinogenicity.^27,28^ A total of 1995 additional alkylated PAHs and PACs containing heteroatoms (N, S, O) were included in the ANST/POL list (Table S2B) but had low reporting frequency. Major alkanes were methane, hexadecane, tetradecane, heptadecane, and pentadecane (Table S4). Similar to PAH/PACs, 570 other alkane/enes (including cyclic alkanes/alkenes without heteroatoms) were reported but at much lower frequency. These alkane/enes were also present in the SUSDAT list and in lists of compounds identified in consumer plastics.^29^ The percent of alkane/enes of total CASRN counts doubled from 0.8% in 1985 to 1.6% in 2015.

Measurements of gases and volatile organic compounds (VOCs) generally increased as a percentage of total CASRNs counts from the late 1960s to 1997 (7.2%), declining to 5.5% by 2021 (Figure 4D). Major compounds were nitrogen oxide, carbon dioxide, ammonia, hydrogen, and carbon monoxide. Benzene and polar organics 3-methyl-1-butanol and 2-methyl-1-propanol were the most prominent organics. The volatile, neutral alkane/enes (methane, ethane) were included with alkane/enes (Figure 4D), while chlorofluorocarbons and hydrofluorocarbons (CFCs/HFCs) were included with PFASs (Figure 4G).

Reports of inorganic ions in water increased sharply over the period 2005–2017 (Figure S2E). However, they represented a declining proportion of total CASRNs counts per year (Figure 4E) dropping from 2.9% in 1966 to 1.1% in 2006 as a greater number of other chemicals were reported. Phosphate and nitrate predominated (43% and 42% of inorganic counts, respectively) followed by sulfate (15%) and perchlorate (0.4%). Twenty inorganic acids and salts were screened separately from the major ions although there was potentially overlap as the most frequently reported analytes were salts related to nitric acid and phosphoric acid. Overall the inorganics ranged from 0.1 to 0.3% of total CASRNs from 1966 to 2021 with no clear trend (Figure 4E).

Disinfection byproducts were identified with the USEPA CompTox Chemicals Dashboard list (Table S1; after editing to avoid duplication with other classes). Among the top 10 compounds were organohalogens, iodoacetic acid, chlorodibromomethane, and 1,3-dichlorobenzene, as well as several oxidation products, phenylacetaldehyde, pentanal, and heptanoic acid. These latter products may have nondisinfection sources as well. Overall, the disinfection byproducts ranged from 0.2 to 0.5% of total CASRNs counts per year, and the number of reports tripled from 363 to 1098 over the period 1990 to 2015 (Figure S2E).

Metals/metalloids and metal isotopes including radioisotopes constituted the third largest of the 20 classes in terms of counts of CASRNs per year (Figure S2-F). As discussed previously, metals were among the top 10 reported substances. In addition to the 5 mentioned above, nickel, chromium, mercury, and selenium were in the top 20 CASRNs reported (Table S4). This high number of reports each year reflects their priority under various monitoring programs for both human and wildlife exposure. However, as a percentage of all CASRNs, metals/metalloids and metal isotopes have declined from 19% in 1980 to 9% in 2021 reflecting the growth of other analytes. Reports of toxic metals cadmium and lead declined slowly from 1980 to 2021 from about 1.4 to 0.9% of total CASRNs, and mercury also declined from 1.1 to 0.4% (Figure 4F).

Poly/perfluoroalkyl substance (PFAS) related compounds were screened with the USEPA “PFASmaster list” of 5076 CASRNs. A total of 234 CASRNs were reported although many were precursors of C_2_ to C_18_ perfluorocarboxylates (PFCAs) and C_4_ to C_12_ perfluoroalkylsulfonates (PFSAs). PFOS, perfluorooctanoic acid (PFOA), and trifluoroacetic acid (TFA) were the top 3 PFASs accounting for 17.7%, 13.5%, and 5.6% of total PFAS counts, respectively, in 2021. PFASs increased sharply from 0.05% of total CASRNs counts in 2001 to 1.2% in 2021 (Figure 4G). The first reports of perfluorooctanesulfonate (PFOS) in environmental media occurred in 2001.^30^ Reports of PFOS and closely related products such as perfluorooctane-sulfonamido compounds accounted for up to 32% of the C_4_–C_12_ PFSAs and C_5_–C_16_ PFCAs (Figure S6). The short chain PFASs (C_2_–C_4_) which are degradates of compounds with C_2_–C_4_ perfluoroalkyl moieties increased from 0.01% in 1970 to 0.06%, of all CASRN reports, in 2021. The major component of this class was TFA which accounted for 100% of the C_2_–C_4_-PFCAs in 1970 and 51% in 2020. The predominance of TFA reflects its importance as a terminal environmental degradation product for many CFCs and HCFCs.^31^

The C1 to C4 hydrofluorocarbons (HFCs), chlorofluorocarbons (CFCs), and hydrochlorofluorocarbons (HCFCs) were screened separately from other PFASs due to their different physical chemical properties and uses as refrigerants and blowing agents. Trichlorofluoromethane, dichlorodifluoromethane, and 1,1,1,2-tetrafluoroethane were the top 3 of the 52 CFCs/HFCs/HCFCs reported. The CFCs/HCFCs/HFCs represented 0.22% of total CASRNs counts per year (Figure 4G) in 1975, declining to 0.008% in 2021. However, maximum counts for CFCs/HCFCs/HFCs CASRNs were achieved in 2005. The reports in the 1970s reflect the discovery of the ozone depleting potential of CFCs/HCFCs and their initial atmospheric measurements.^32,33^

Fragrance chemicals represented a significant proportion of total CASRN counts, ranging from 2.8 to 9.5% (Figure 4H). The top 10 fragrances were all terpenoids with beta-caryophyllene and beta-pinene being most reported. These substances are used in fragrances but are used in many other products including essential oils and as food additives. In addition, they are natural products emitted by terrestrial vegetation,^34^ and the large number of reports reflects, in part, the extensive environmental measurements. However, the large number of reports for fragrances relative to PAHs and plastics additives may be an indication that the BIOL/occur also captured reports other than environmental measurements.

Plastics additives were identified based on the recent list of more than 10,000 substances published by Wiesinger et al.^35^ Duplicates related to other classes were removed leaving 8296 CASRNs (Table S1). This diverse list of compounds included 93 phthalates and 80 dyes and pigments. Reports of these compounds grew 3-fold from 2002 to 2014 (Figure S2H) although in terms of the proportion of all CASRNs the range was from 2.5 to 2.9% (Figure 4H). The potential environmental contamination from additives in polymers has had a growing number of studies as part of the larger topic of plastic litter and microplastics.^36^ Bisphenols and alkyl phenols also represent a large class of plastics additives but are considered separately below.

Food additive chemicals represented 0.8 to 1.3% of total CASRN counts (Figure 4H). This group of 103 CASRNs was a subset of a much larger array of 922 compounds in the initial ANST/POL list Poll which we considered natural products/constituents. The top 5 chemicals were 4 substances used as flavors or scents, benzene acetaldehyde, scopolamine, trimethylbenzyl alcohol, dimethyl disulfide, and 1-naphthylacetic acid, a plant growth regulator and food contaminant.^19^ As noted for pharmaceuticals, the larger array of food additives captured in the ANST/POL list is likely due to inclusion of an extensive number of food chemistry related studies which involve analysis although not of environmental media.

Environmental measurements of phenolic antioxidant additives in plastics, the bisphenols^37,38^ and the hindered alkyl phenols,^39^ increased sharply, doubling from 2014 to 2021 (Figure S2-I). The bisphenol/alkyl phenols increased as a percent of total CASRNs counts per year from 0.2% in 2000 to 0.5% in 2021 (Figure 4I). Bisphenol A (BPA) and bisphenol S were the most prominent bisphenols, while butylated hydroxytoluene (BHT), phloretin, and 4-*tert*-butylphenol were the most widely measured alkyl phenols. A previous review showed that BPA was the most widely measured with over 500 articles on its environmental fate and distribution published by 2015.^40^ The prominence of BHT likely reflects its use in food packaging as well as consumer product and industrial applications,^39^ while the presence of phloretin, a natural phenolic compound, may reflect efforts to analyze it in foods and biological fluids.^41^

Cyclic alkyl- and alkyl/aryl siloxanes and silanes represented a relatively small but growing subset of measurements of emerging chemicals rising from 0.01% in 1990 to 0.28% in 2014 (Figure 4I). The major compounds were the cyclic methyl siloxanes (D3, D4, D5, and D6) accounting for 51% of all measurements within this class. The cyclic methyl siloxanes are high production volume chemicals used to make polydimethyl silicone polymers as well as direct use in consumer products.^42,43^ Several alkylsilanes were also included in the ANST/POL list; however, their presence may be due to use during environmental analyses, e.g. octadecyl silane is used for reversed phase chromatography materials.

Benzotriazoles (BZTs) are widely used as anticorrosion agents and as UV filters in personal care products and polymers.^44,45^ Benzothiazoles (BTs) and aromatic amines (AAs) are synthetic rubber additives.^46^ AAs are also widely used as dyes^47^ and can be released from degradation of azo-dyes. The number of reports of these classes has increased steadily from the 1990s to 2021 (Figure S2-I). However, as a percent of total CASRNs reported each year, the BZT/BT/AA group has declined from 0.2% to 0.08% from 1990 to 2021 (Figure 4). Recent interest in the fate and toxicity chemicals associated with tire wear particles^48,49^ may increase the number of reports for these substances. 1,1′-Biphenyl-4-amine, 4,4′-Diaminobiphenylmethane, and 4-methyl-1,3-Benzenediamine were the most commonly reported compounds in this class possibly reflecting measurements related to concerns over human exposure.^50^

### 4 Overlap of Environmental Occurrence Data and Industrial Chemical Inventories

**Figure 5:**
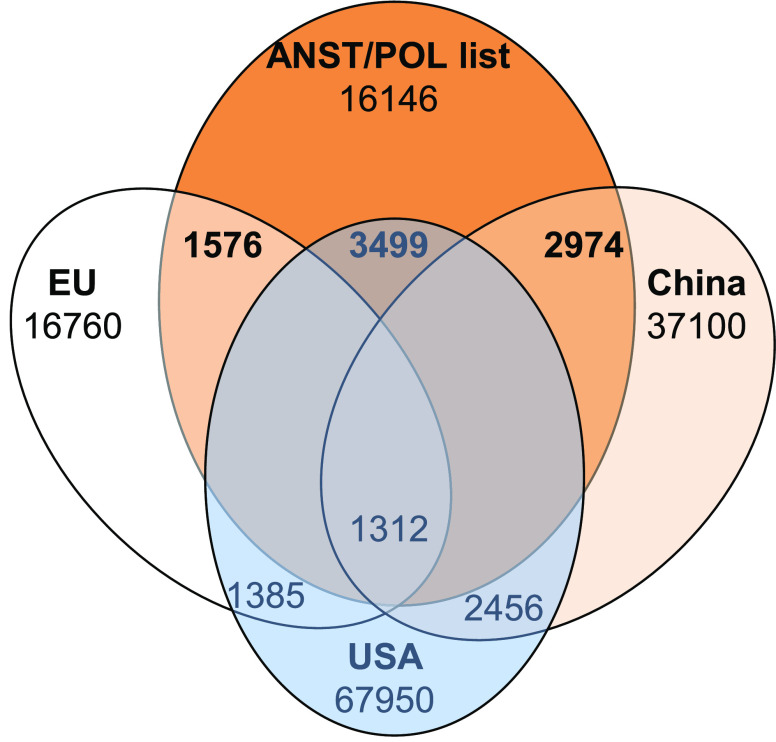
Venn diagram showing the overlap (upper row of numbers) in CASRNs between the publicly available industrial chemical inventories (USA, (TSCA, 67,950 CASRNs), EU - REACH inventory of registered substances (16,760), and China (IECSC; 37,100)), with the ANST/POL list from the CAplus database. The lower row of numbers represents the overlap of the CASRNs within each inventory. Pesticides and pharmaceuticals were not included as they are generally not listed in the TSCA, REACH, or IECSC inventories.

Many of the substances in the ANST/POL list are industrial chemicals, and therefore, we were interested to assess progress in measurements of their environmental occurrence. We compared the CASRNs in the ANST/POL list with the industrial chemical inventories of the US, EU, and China. These 3 regions/countries represent the largest manufacturing and use areas for chemicals globally^4,51^ as well as the regions where most environmental measurements have been made. The inventories were assembled by downloading publicly available lists of substances (Table S6). Because these inventories generally do not contain pesticides or pharmaceuticals, as those classes are under separate regulatory legislation, we removed them from the ANST/POL list, yielding 16146 CASRNs. The overlap of CASRNs from the US, EU, and China inventories with the ANST/POL list is illustrated in Figure 5. The US TSCA inventory had the largest number (3499), followed by China’s IECSC with 2974, and the EU REACH inventory with 1576, representing 5.1, 7.5, and 9.4% of the inventories, respectively. The results reflect, in part, the size of the inventory and also the time period over which the inventory has been developed. TSCA is the largest of the three (Table S6) and includes substances added in the 1980s, while the REACH inventory is the most recent having been initiated in 2007.^52^ After removing duplicates, 4253 CASRNs or 4.8%, in the ANST/POL list, were present in the combined inventories with the pesticides and pharmaceuticals excluded. The list is included in Table S3. The limited overlap reflects the presence of numerous individual chemicals, e.g. PCB and PBDE congeners and their transformation products, among the classes in the ANST/POL list, as shown in Table S2B, whereas about 30% of the substances on inventory lists are mixtures or UVCBs (unknown or variable composition, complex reaction products, or biological materials).^4^

**Figure 6:**
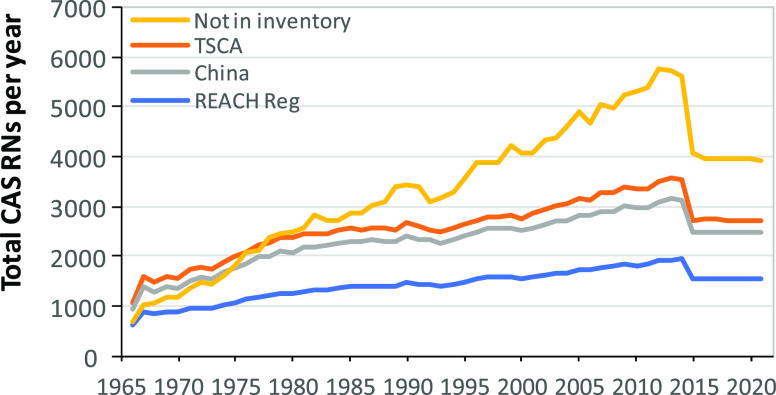
Trends over time of citation counts of CASRNs in the industrial chemical inventories of the USA, European Union, and Peoples Republic of China (TSCA, REACH, and IECSC) based on the CASRNs reported in the BIOL/Occur search. Trends for citation counts of CASRNs which were not present in these 3 inventories are also shown.

We were also interested in temporal trends of measurements of the industrial chemicals with environmental measurements. Figure 6 depicts the trends of the CASRNs in the China, EU, and US inventories over time based on citations in the BIOL/occur list. The overall numbers increased gradually from the 1960s consistent with the general overall increase of the total number of CASRNs (Figure 1). Many of these CASRNs were undergoing environmental measurements long before being added to the chemical inventories. The trends reflect the continued measurements of a relatively narrow list of chemicals as observed for the full ANST/POL list (Figure 2) along with the expansion of interest in new classes of contaminants beyond those considered under chemical legislation. The sharp decline in 2015–2018 is likely due to the fact that the ANST/POL list was created in 2018, and thus, no newly reported substances were included in the BIOL/occur for 2019–2021. Given the relatively low proportion of these CASRNs in the three inventories being reported, the results suggest that presence on a chemical inventory list is not a major driver of targeted environmental measurement.

### 5 Implications

This study has shown that a large number of substances have been measured in environmental media over the past 50 years. The precise number is difficult to determine given the multiple ways measurements are referred to in the literature, e.g. biomedical and food based analyses overlap those for environmental media. Also indexing of CASRNs in terms of the roles of analysis or as pollutant may have evolved over this long time period. Nevertheless, given the number of citation counts (9.251 × 10^6^) of the 19,776 CASRNs in our final list, a search using the CAplus database was the only practical and effective approach to accomplishing our goals.

The number of new CASRNs reported using the ANST/POL search increased into the mid-1990s coinciding with the rapid development of HPLC-MS technologies for expansion of analytical scope into polar and ionizable substances and then in the 2000s with the increased uses of high-resolution accurate-mass mass spectrometry to measure pollutants in engineered and natural systems. Increasing the analytical window accessible to environmental chemists has led to increased identification rates for so-called “known-unknowns”. Namely, chemicals widely used in commerce but that were difficult to measure by GC-MS became analytically accessible in this period and were readily identified. This list includes some PFAS chemicals, fragrances, bisphenols/alkyl phenols, and pharmaceuticals (Figure 4). The results of our search illustrate that when chemical standards, molecular databases, and mass spectral libraries become generally available and integrated with advanced analytical chemistry techniques, rapid progress in the identification and measurement of various classes in environmental media occurs, as illustrated for total CASRN counts for current use pesticides, pharmaceuticals, PFCAs, PFSAs, and bisphenols/hindered phenols over the period 2000–2021 (Figures 4 and S2). As pointed out by Hites,^17^ expansion of the number of substances being analyzed is closely tied to increases in information on toxicity and environmental persistence of various classes of substances. However, despite huge improvements in analytical instrumentation and methodology over the 50 year period of this review, the majority of chemicals in global industrial chemical inventories has not been analyzed in environmental media. Lack of information on environmental toxicity, persistence, and transformation of the majority of industrial chemicals^53^ is thus a major factor influencing progress in detecting, annotating, and quantifying environmental contaminants.

Our search of CASRNs has also confirmed previous findings that a significant portion of all reported environmental chemical measurements are accounted for by a relatively small number of substances; the top 100 accounted for 34% of the 19,776 substances in the ANST/POL list. While Grandjean et al.^11^ reached a similar conclusion based on a detailed survey of the literature for 2000–2009, our study shows that this focus has persisted since the mid-1970s when lists of priority chemicals and exposure guidelines were developed. For example, the 16 PAH Priority Pollutants were listed in the early 1970s,^54^ and the “Persistent Toxic Substances” and “Hazardous Polluting Substances” in the Great Lakes Water Quality Agreement were established by 1978.^55^ Many other countries established similar lists during that time. More recent lists, e.g. the Stockholm Convention list of POPs^56^ and the EU Directive on Environmental Quality Standards,^57^ have also had a great influence in terms of selection of analytes and commercial availability of standards. Halden^15^ referred to the regulatory influence on the publication activity for individual priority chemicals. We also saw some evidence for this with the legacy OC pesticides (Figure S2B) which, like DDT in Halden’s example, had a large number of publications in the 1970s and in the period 2005–2015. If industrial chemical regulations under TSCA, REACH, IECSC, and other inventories required manufacturers to make available analytical standards of new and existing chemicals in commerce, this would eventually go a long way for broadening the number of chemicals that could be measured. Efforts that might have been spent “discovering” new substances could instead be invested in monitoring and environmental fate/effects studies. A publicly accessible global inventory of all chemicals on the market, with associated production and sales data as recommended by Wang et al.,^4^ would also help to stimulate analytical efforts.

While nontarget screening has been conducted in some form, along with targeted analyses, since environmental chemists gained access to mass spectrometers,^17^ recent efforts have focused on leveraging informatics and algorithms to lower the required effort to make confident structure assignments from mass spectrometric data.^2^ It follows that a key step to increasing the number of chemicals and transformation products being measured in the environment would be to make nontarget screening a routine component of environmental chemical monitoring programs, complementing targeted analyses of priority chemicals.^3^ Making this a practical endeavor will require streamlined incorporation of regulatory lists into nontarget screening workflows, ideally with compound-specific metadata (e.g., annual production and use quantities) for use in prioritization. Automated and accurate biotransformation prediction tools could also be incorporated to improve annotation and measurement of environmental degradants not present on regulatory lists. By broadening access to sophisticated analytical techniques capable of facilitating identification of new environmental contaminants, more researchers can join in the search for new contaminants, which should limit the influence of the Matthew effect in environmental monitoring studies.

## Supporting Information Available

Further details on the CAS Search and discussion of total CASRN citation reports, Tables S1, S2, S5, and S6 with additional information including the chemical lists/databases used to screen the BIOL/occur search and classes of substances removed from the ANST/POL list, and additional figures, S1 to S6, with further information on trends of citation counts and counts of CASRNs per year (PDF)
Excel files (Tables S3 and S4) with the final ANST/POL list comprising 19,776 CASRNs and the top 500 substances from the ANST/POL and BIOL/Occur searches (XLSX)
