# Supplementary material for: How Many Chemicals in Commerce Have Been Analyzed in Environmental Media? A 50 Year Bibliometric Analysis
Source: Environ Sci Technol. 2023 Jun 15;57(25):9119–29. doi: 10.1021/acs.est.2c09353 (PMC10308830; doi:10.1021/acs.est.2c09353)
Supplement: Supplementary file 1 — es2c09353_si_001.pdf [file es2c09353_si_001.pdf]

Supporting information

How Many Chemicals in Commerce Have Been Analysed in Environmental Media? A 50 year Bibliometric Analysis

Derek C.G. Muir[1,2*], Gordon J. Getzinger[3,4], Matt McBride[5], P. Lee Ferguson[4]

[1]Environment & Climate Change Canada, Burlington, ON L7S1A1 Canada

[2]School of Environmental Sciences, University of Guelph, Guelph ON N1G2W1 Canada

[3]School of Environmental Sustainability, Loyal University Chicago, Chicago IL 60660 USA

[4]Department of Civil and Environmental Engineering, Duke University, Durham NC 27708 USA

[5]CAS IP services, CAS, Columbus OH 43202 USA

*Corresponding author : derek.muir@sympatico.gc.ca

Summary : 15 pages, 6 Tables, 6 Figures

Table of Contents

Text	page
1. Further details on the CAS Search<br>2. Further discussion of Table 1	S3
Tables	
Table S1. List of chemical lists/databases used to screen the BIOL/occur search	S4
Table S2. A. Classes of substances removed from the ANST/POL list<br><br>Table S2. B. Classes of substances in the ANST/POL list and illustrated in Figure 3	S7
Table S3. Final ANST/POL list comprising 19,776 CASRNs including common name, InChl, SMILES, and Category where available (Separate Excel file: Muir et al Table S3 & S4.xlsx)	S9
Table S4. Top 500 substances from the ANST/POL and BIOL/Occur searches (Separate Excel file: Muir et al Table S3 & S4.xlsx))	S9

Table S5. Comparison of reports for the top 5 pharmaceuticals in environmental samples	S10
Table S6. Information related to the inventories of United States, Peoples Republic of China, and the European Union	S11
Figures	
Figure S1. (A). Total counts of CASRNs per year for the 19,776 ANST/POL list and for 10,695 CASRNs identified from various priority and monitoring lists. (B) Percent that the 10,695 substances on lists as well as the 3041 substances on the Meijer et al/SUSDAT CEC list represent are also shown.	S12
Figure S2. Trends in total citation counts of CASRNs reported each year for (A) pharmaceuticals, current use pesticides, and CECs not on other lists, (B) Legacy organochlorine (OC) pesticides, polychlorinated dibenzo-p-dioxins and –dibenzofurans (PCDD/Fs), polychlorinated biphenyls (PCBs), (C) polybrominated diphenyl ethers (PBDEs), organophosphate flame retardants and phosphate esters (OPFR-OPEs), other flame retardants (FRs), (D) Volatile organic compounds and trace gases, polycyclic aromatic hydrocarbons/compounds (PAHs/PACs), alkane/enes, (E) Major water pollutant ions, inorganic acids, disinfection byproducts, (F) Metal isotopes and major metals (scale on the right), copper (Cu), magnesium (Mg), cadmium (Cd), lead (Pb) and mercury (Hg), (G) poly/perfluoroalkyl substances (PFAS) including chlorofluorocarbons and hydrofluorocarbons (CFC-HFCs), (H) Food additives, plastics additives, fragrances, (I) Bisphenols, siloxane/silanes, aromatic amines, benzotriazoles/benzothioazoles. The CASRNs also include transformation products for all groups.	S12
Figure S3. Trends in total citation counts of CASRNs reported each year for (A) 1809 pharmaceuticals and transformation products in the BIOL/occur list and for the 661 substances in the UBA database of pharmaceuticals and related compounds measured in environmental media and present in the ANST/POL list. (B) Percent of pharmaceuticals and the UBA database substances represented of total CASRN counts in the BIOL/occur list.	S13
Figure S4. Trends in (A) total counts of CASRNs and (B) percent of CASRN counts, reported each year for halogenated aromatic compounds and other non-halogenated aromatic compounds in the list of 3041 Chemicals of Emerging Concern (CECs) from the NORMAN SUSDAT list and Meijer et al. [1].	S13
Figure S5. Trends in (A) total counts of CASRNs and (B) percent of CASRN counts, reported each year for flame retardants with (1) no chlorine, bromine or phosphorus, (2) chlorinated flame retardants (PCDEs, chlorinated polycyclic aromatics, chlorinated paraffins, mirex, Dechlorane) and (3) brominated flame retardants other than PBDEs	S13

Figure S6. Trends of (A) Total counts of CASRNs for PFOS, TFA and PFOA-related compounds and (B) Percent of CASRN counts of total PFAS (Figure 4G) based on 234 PFAS identified from the USEPA PFASMaster list.	S14
References	S15

1. Further details on the CAS Search

We developed a targeted CAS Role search for substances associated in citations reported in analytical studies (ANST) and as a pollutant (POL). It can be referred to as a 'linked' CAS Role search. CAS roles are CAS indexing terms consisting of codes that describe the new or novel information reported about a substance or a class of compounds

"POL" or "Pollutant": Defined by Chemical Abstracts (Vol. 66, 1967 to present): Assigned to a substance in studies in which the substance (viewed as a harmful substance) is encountered in the environment, including indoor and outdoor air and atmosphere, soil, water, buildings, biological systems, etc. This role is also used for substances in studies which focus on potentially harmful effects if the substance were to enter an ecosystem. In analysis for pollutants in environmental samples, the pollutant-analyte receives ANST and POL.

"ANST" or "Analytical Study". Defined by Chemical Abstracts (Vol. 66, 1967 to present): Assigned to a substance/material in studies of the detection or identification of the constituents of the material; of the determination of the amount of a constituent in the material; of qualitative or quantitative bioassays; of involvement of the substance in an analytical procedure; for separation of the substance with analytical intent; or for identification of an unknown substance. ANST roles are not assigned to the substance when a routine analytical procedure is used as a tool to verify results of a reaction or process.

"BIOL or Biological Study/RL. Defined by Chemical Abstracts (Vol. 66 1967 to present): Assigned to a substance in studies of the role of the substance in or its effect on biological molecules and systems (including organisms, organs, cells, and subcellular systems). Such studies include metabolism, toxicity, occurrence, biological applications and composition.

We used CAS STN (About CAS STNext® | STN International (stn-international.com) to search the CAS Content Collection. Unfortunately, this search approach cannot be performed/duplicated in CAS SciFinder, although the same database content is available on both platforms. However the search functionality is not equivalent. STN allows users to start at the CAS Role level for substances without searching for citation concepts. Our approach was to isolate substances with the appropriate linked roles first.

2. Further discussion of Table 1

"Total CASRN citation reports": Initially 105,410 citations were identified that contained the dual substance role indexing of ANALYTICAL STUDY and POLLUTANT. The Report citation count represents the number of citations where the identified CAS RNs are present. That resulted in an initial list of 23,458 unique CAS RNs associated with those roles in those citations. "Total CASRN citation counts" represents the total number of substance hits across those citations. A single citation may have 8 substances associated with those roles, leading to a count of 8 for CASRN Count. A substance would only be counted once in each citation, but a citation may have multiple qualifying substances.

Table S1. List of chemical lists/databases used to screen the BIOL/occur search

Name	Source	Number of Chemicals	Notes
Pesticides	EPA "PESTICIDES" list from Office of Pesticide Programs Information Network, USEPA CompTox Chemicals Dashboard. Transformation products from the ANST/POL list were added	4031	Pharmaceuticals and legacy pesticides removed;
Legacy pesticides	Includes DDT-related, chlordanes, toxaphenes, hexachlorocyclohexanes, dieldrin/endrin, pentachlorophenol, +20 butyl tins (from Anal/PollANST/POL search)	147	Transferred from Pesticides list and identified in ANST/POL search
Pharmaceuticals	Combined lists from Howard & Muir [2], ZINC15PHARMA, Swiss/US list and DrugBank list from USEPA CompTox Chemicals Dashboard	7383	Duplicates among the 3 lists removed. Pesticides removed
IFRA Transparency List	International Fragrance Association https://ifrafragrance.org/initiatives/transparency/ifra-transparency-list	3024	Pesticides, pharmaceuticals and PAHs removed
Bisphenols	"NORMAN|LIST" from USEPA Chemicals Dashboard + list of 20 hindered phenols	73	additional CASRNs added
PAHs and related polycyclics + n-alkanes	"PAHLIST" from USEPA CompTox Chemicals Dashboard and Supporting Information table in Achten and Andersson [3] + others from ANST/POL list	1325	Included n-alkanes, nitro and oxy-PAHs, and heterocyclics
Flame retardants	Included chlorinated paraffins from "C10CHLOROPARAFF" and "FLAMERETARD" from USEPA CompTox Chemicals Dashboard. Also included PBBs, PCDEs, bromophenols, bromo/chloro-benzenes from ANST/POL search	879	PBDEs removed.
PBDEs	"PBDES" List from USEPA CompTox Chemicals Dashboard + other PBDEs in the ANST/POL search.	246	Including PBDE metabolites, bromo-chloro BDEs and

			alternate CASRNs
PCBs	"PCBCHEMICALS" List from USEPA CompTox Chemicals Dashboard + PCB transformation products from ANST/POL list	429	Including 211 PCB metabolites and alternate CASRNs
PFAS	"PFASMASTER" List of PFAS Substances from USEPA CompTox Chemicals Dashboard	5076	Used without editing
Elements and major ions	From ANST/POL search. Identified by common name and InChI code	728	Includes metal isotopes and radionuclides
atmospheric trace gases	From ANST/POL search. Identified by common name and InChI code (267). Includes CFC/HFCs (165)	432	
Disinfection byproducts	Disinfection By-products (Richardson et al)<br><br>(USEPA CompTox Chemicals Dashboard)	533	Pesticides, fragrances, pharmaceuticals and flame retardants removed
Benzotriazole/thiazoles and aromatic amines	AROMATICAMINES (22), TIRECRUMB (290) USEPA CompTox Chemicals Dashboard	312	Removed duplicates with plastic additives
Food additives and natural constituents	FOODB from Wishart lab – https://www.wishartlab.com/web_servers	15948	pesticides, pharmaceuticals and fragrances removed
Plastics additives	PlasticsAdd list published by Wiesinger et al. [4]	8296	Pesticides, PAH, alkane/enes, bisphenols, hindered phenols,

			pharmaceuticals and fragrances removed
Chemicals of Emerging Concern (CECs)	NORMAN Suspect List Exchange (http://www.norman-network.com/?q=node/236)<br><br>Accessed August 2020 via USEPA CompTox Chemicals Dashboard	62,259	Wide range of chemistries plus predicted Phase 1 metabolites
Volatile organic compounds (VOCs)	VocBinBase volatile compounds database (USEPA CompTox Chemicals Dashboard); Supplementary Table in Wang et al. [5]; List from Condorchem Inc https://condorchem.com/en/ volatile-organic-compound-list/	268	Duplicates removed
Transformation products.	NORMAN: Parent-Transformation Product Pairs from EAWAG (USEPA CompTox Chemicals Dashboard) + 30 additional substances from ANST/POL list	262	Some duplication with pesticide list
Mycotoxins	MYCOTOX2 LIST: Mycotoxins (USEPA CompTox Chemicals Dashboard)	328	Screened ANST/POL list and removed

Table S2. A. Classes of substances removed from the ANST/POL list

Class	Original list	After editing
Acid	6	0
Alloy	18	0
Complex structure	116	0
Mass labelled	117	0
Mineral	138	0
Inorganic salts	902	0
Polymer	157	0
Elements + Metal Isotopes	887	836
Food related	953	103
Natural product	1082	0
No structure	78	0
Unknown structure	13	0
Enzymes	15	0
Mixture	69	0
All others		18837
With InchI codes	23395	19740
With SMILES	17600	15175

Table S2. B. Classes of substances in the ANST/POL list and illustrated in Figure 3

Class	Number of CASRNs	%
Pharmaceuticals	1809	9.15%
Br, Cl and P related flame retardants[1]	1417	7.17%
PCB related	453	2.29%
PBDE related	170	0.86%
PFAS related	295	1.49%
PCDD/Fs	322	1.63%

Siloxane/silanes	74	0.37%
Plastics additives	784	3.96%
Bisphenols+ alkyl phenols	46	0.23%
Benzotriazole/thiazoles and aromatic amines	68	0.34%
In use pesticides	1821	9.21%
Legacy pesticides	125	0.63%
Metals/isotopes	836	4.23%
Major ions+inorganic acids	26	0.13%
Gases/VOC/CFC/HFCs	183	0.93%
Alkane/enes	575	2.91%
PAH/PACs	1215	6.14%
Fragrances	566	2.86%
Disinfection byproducts	146	0.74%
Food related	103	0.52%
Organometallics	77	0.39%
CECs from Meijer et al. (2020)/ NORMAN network SUSDAT list	3041	15.4%
Not characterized	5624	28.4%

[1]Includes BFRs, chlorinated paraffins, polychlorinated polyaromatics,

[2]Includes products identified using CompTox Chemicals Dashboard lists. Most transformation products were identified manually and included with each substance group

Table S3. Final ANST/POL list comprising 19,776 CASRNs including common name, InChl, SMILES, and Category where available (Separate Excel file: Muir et al Table S3 & S4.xlsx)

Table S4. Top 500 substances from the ANST/POL and BIOL/Occur searches (separate Excel file: Muir et al Table S3 & S4.xlsx)

Table S5. Comparison of reports for the top 5 pharmaceuticals in environmental samples

Top Five		CASRN	UBA reports[1]	ANST/POL list BIOL/Occur search[2]	SciFinder Occurrence role[3]	Scifinder Biological role[4]
UBA database	ibuprofen	15687-27-1	6,848	940	2204	
	Diclofenac	15307-86-5	6,491	5958	2874	
	Carbamazepine	298-46-4	6,323	8241	2799	
	Sulfamethoxazole	723-46-6	5,397	15228	3127	
	Trimethoprim	738-70-5	3,976	16600	1793	
BIOL/Occur search	Cisplatin	15663-27-1	NA	38929	62	
	Dexamethasone	50-02-2	90	34991	205	
	Ciprofloxacin	85721-33-1	2064	33724	2292	
	Gentamicin	1403-66-3	6	32825	440	176
	Doxorubicin	23214-92-8	17	31301	44	31

[1]Number of reports or studies in the UBA database for each chemical

[2]Number of reports or studies from the CASPlus database based on reduced list of 19800 from the ANST/POL and then search using roles of BIOL and Occurrence

[3] SciFinder search for each CASRN via "substance identifier" then selecting the Role "Occurrence", refining for period 1960-2018, and then categorizing "environmental chemistry", "pollutant"

[4] SciFinder search for each CASRN via "substance identifier" then selecting the Role "Biological", refining for period 1960-2018, and then categorizing by "environmental chemistry", "pollutant"

Table S6. Information related to the inventories of United States, Peoples Republic of China, and the European Union

Country or Region	Inventory	Web address	Number of CASRNs[1]
USA	Toxic Substances Control Act (TSCA)	https://www.epa.gov/tsca-inventory/how-access-tsca-inventory (accessed June 2018)	67950
China	Inventory of Existing Chemical Substances of China (IECSC)	http://www.mee.gov.cn/gkml/hbb/bgg/201301/t20130131_245810.htm (accessed March 2017)	37107
European Union	REACH inventory – registered substances	https://echa.europa.eu/information-on-chemicals/registered-substances (accessed January 2019)	16760

[1]Number of substances in downloadable files with CASRNs

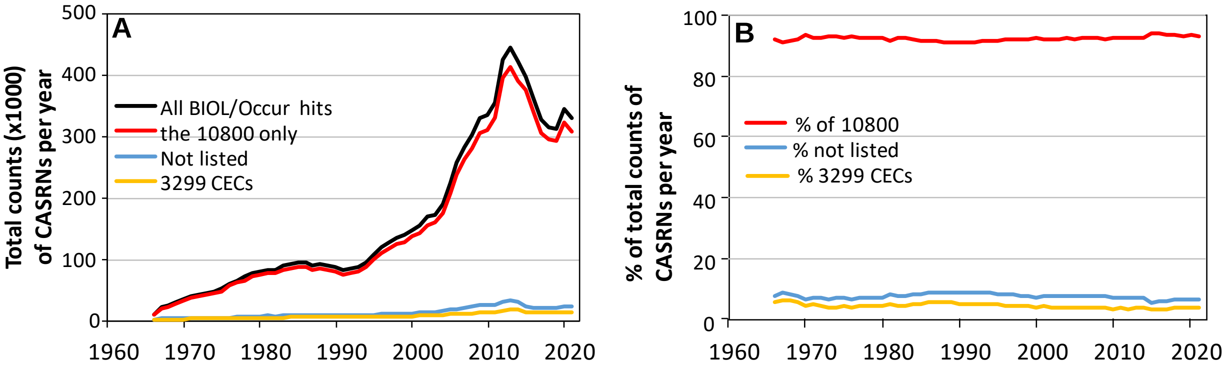

Figure S1. (A). Total counts of CASRNs per year for the BIOL/occur search using the ANST/POL list and for 10,800 CASRNs identified from various priority and monitoring lists. (B) Percent that the 10,800 substances on lists as well as the 3041 substances on the Meijer et al/SUSDAT chemicals of emerging concern (CEC) list represent are also shown.

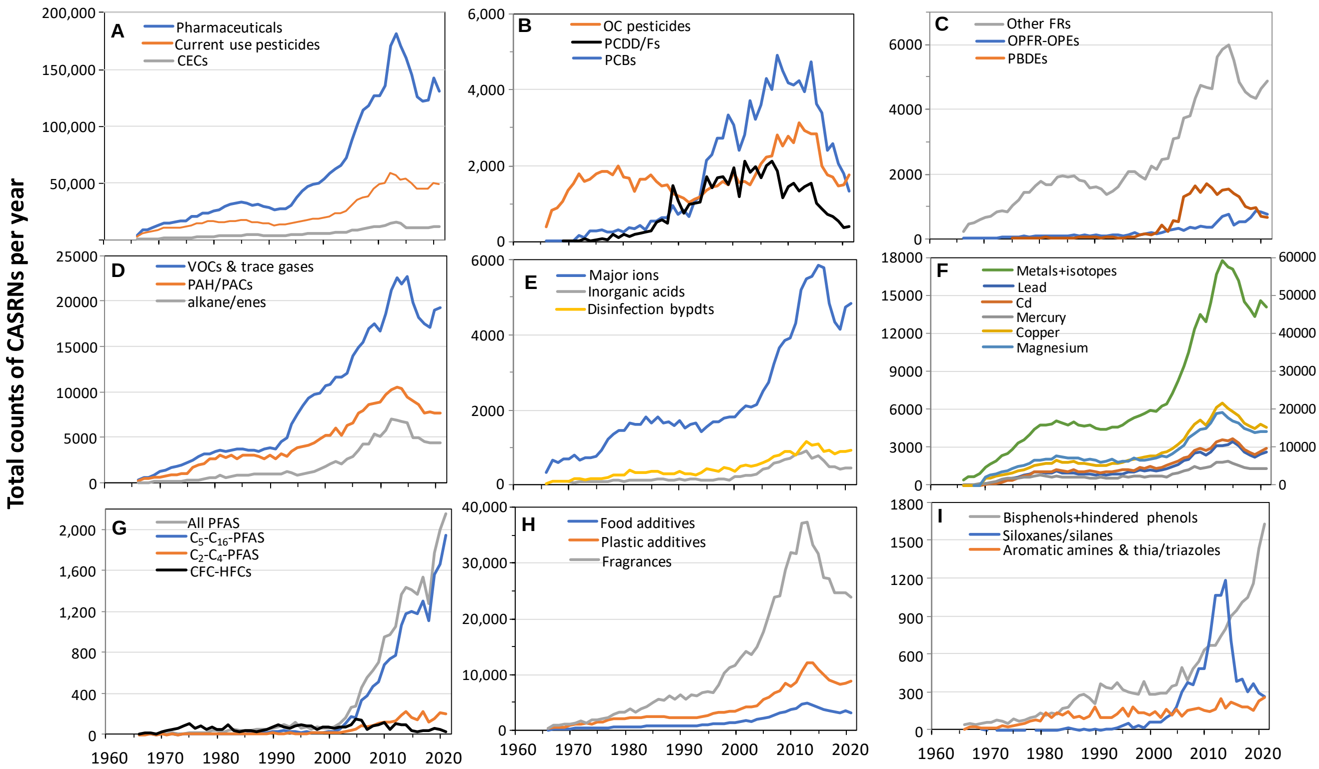

Figure S2. Trends in total citation counts of CASRNs reported each year for (A) pharmaceuticals, current use pesticides, and CECs not on other lists, (B) Legacy organochlorine (OC) pesticides, polychlorinated dibenzo-p-dioxins and –dibenzofurans (PCDD/Fs), polychlorinated biphenyls (PCBs), (C) polybrominated diphenyl ethers (PBDEs), organophosphate flame retardants and phosphate esters (OPFR-OPEs), other flame retardants (FRs), (D) Volatile organic compounds and trace gases, polycyclic aromatic hydrocarbons/compounds (PAHs/PACs), alkane/enes, (E) Major water pollutant ions, inorganic acids, disinfection byproducts, (F) Metal isotopes and major metals (scale on the right), copper (Cu), magnesium (Mg), cadmium (Cd), lead (Pb) and mercury (Hg), (G) poly/perfluoroalkyl substances (PFAS) including chlorofluorocarbons and hydrofluorocarbons (CFC-HFCs), (H) Food additives, plastics additives, fragrances, (I) Bisphenols, siloxane/silanes, aromatic amines, benzotriazoles/benzothioazoles. The CASRNs also include transformation products for all groups.

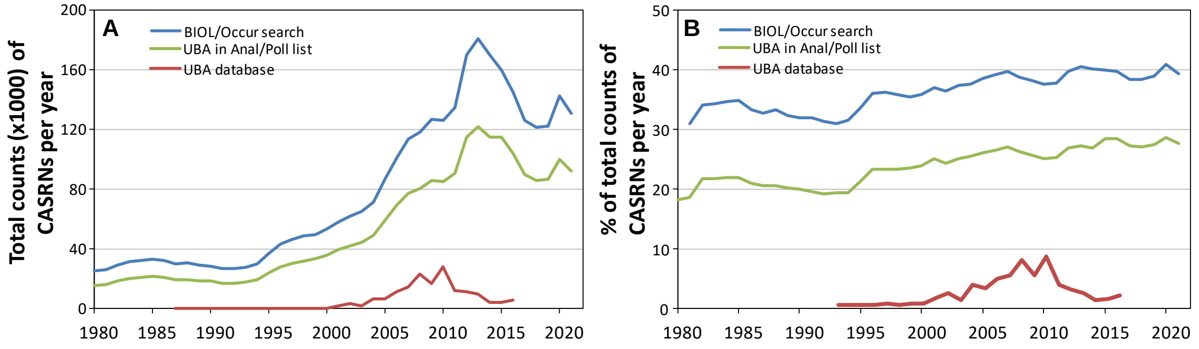

Figure S3. Trends in total citation counts of CASRNs reported each year for (A) 1809 pharmaceuticals and transformation products in the BIOL/occur list and for the 661 substances in the UBA database of pharmaceuticals and related compounds measured in environmental media and present in the ANST/POL list. (B) Percent of pharmaceuticals and the UBA database substances represented of total CASRN counts in the BIOL/occur list.

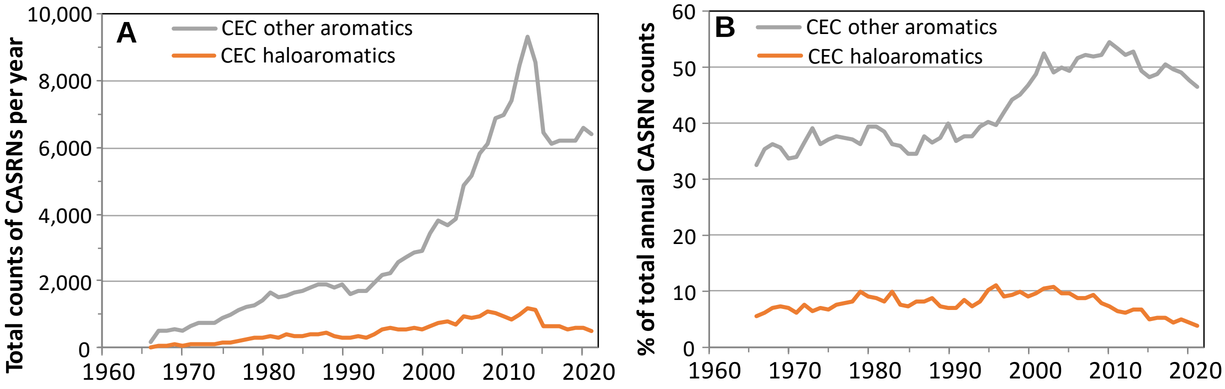

Figure S4. Trends in (A) total citation counts of CASRNs and (B) percent of CASRN counts, reported each year for halogenated aromatic compounds and other non-halogenated aromatic compounds in the list of 3041 Chemicals of Emerging Concern (CECs) from the NORMAN SUSDAT list and Meijer et al. [1].

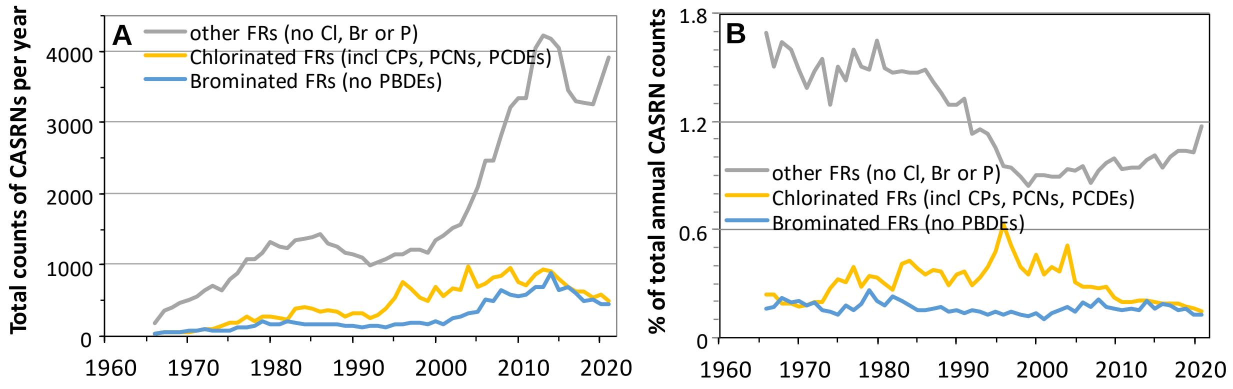

Figure S5. Trends in (A) total citation counts of CASRNs and (B) percent of CASRN counts, reported each year for flame retardants with (1) no chlorine, bromine or phosphorus, (2) chlorinated flame retardants (PCDEs, chlorinated polycyclic aromatics, chlorinated paraffins, mirex, Dechlorane) and (3) brominated flame retardants other than PBDEs

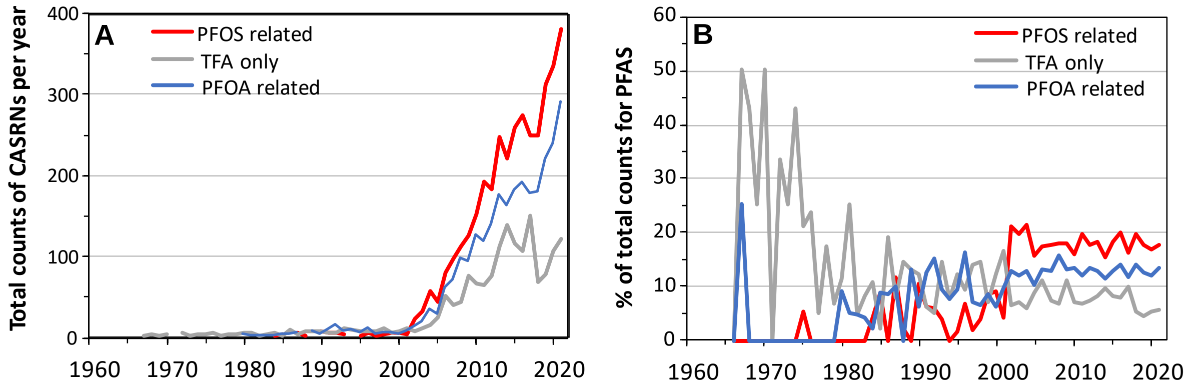

Figure S6. Trends of (A) Total citation counts of CASRNs for PFOS, TFA and PFOA-related compounds and (B) Percent of CASRN counts of total PFAS (Figure 4G) based on 234 PFAS identified from the USEPA PFASMaster list.

References

(1) Meijer, J.; Lamoree, M.; Hamers, T.; Antingac, J.-P.; Hutinet, S.; Debrauwer, L.; Covaci, A.; Huber, C.; Krauss, M.; Walker, D. I.; Schymanski, E.; Vermeulen, R.; Vlaanderen, J. *HBM4EU CECscreen: Screening List for Chemicals of Emerging Concern Plus Metadata and Predicted Phase 1 Metabolites. S71. CECSCREEN.* https://zenodo.org/record/3956587#.X0kY3aRE1aQ; 2020.
(2) Howard, P. H.; Muir, D. C. G. Identifying New Persistent and Bioaccumulative Organics Among Chemicals in Commerce II: Pharmaceuticals. *Environ Sci Technol.* **2011**, *45*, 6938-6946.
(3) Achten, C.; Andersson, J. T. Overview of Polycyclic Aromatic Compounds (PAC). *Polycyclic Aromatic Compounds* **2015**, *35* (2-4), 177-186. DOI: 10.1080/10406638.2014.994071.
(4) Wiesinger, H.; Wang, Z.; Hellweg, S. Deep Dive into Plastic Monomers, Additives, and Processing Aids. *Environmental Science & Technology* **2021**, *55* (13), 9339-9351. DOI: 10.1021/acs.est.1c00976.
(5) Wang, Y.-C.; Lin, C.; Lin, Y.-K.; Wang, Y.-F.; Weng, W.-H.; Kuo, Y.-M. Characteristics and determinants of ambient volatile organic compounds in primary schools. *Environmental Science: Processes & Impacts* **2016**, *18* (11), 1458-1468, 10.1039/C6EM00491A. DOI: 10.1039/C6EM00491A.
